# Supplementary material for: Spin injection and inverse Edelstein effect in the surface states of topological Kondo insulator SmB6
Source: Nat Commun. 2016 Nov 11;7:13485. doi: 10.1038/ncomms13485 (PMC5114616; doi:10.1038/ncomms13485)
Supplement: Supplementary Information — Supplementary Figures 1-5, Supplementary Notes 1-4 and Supplementary References [file ncomms13485-s1.pdf]

Supplementary Figure 1

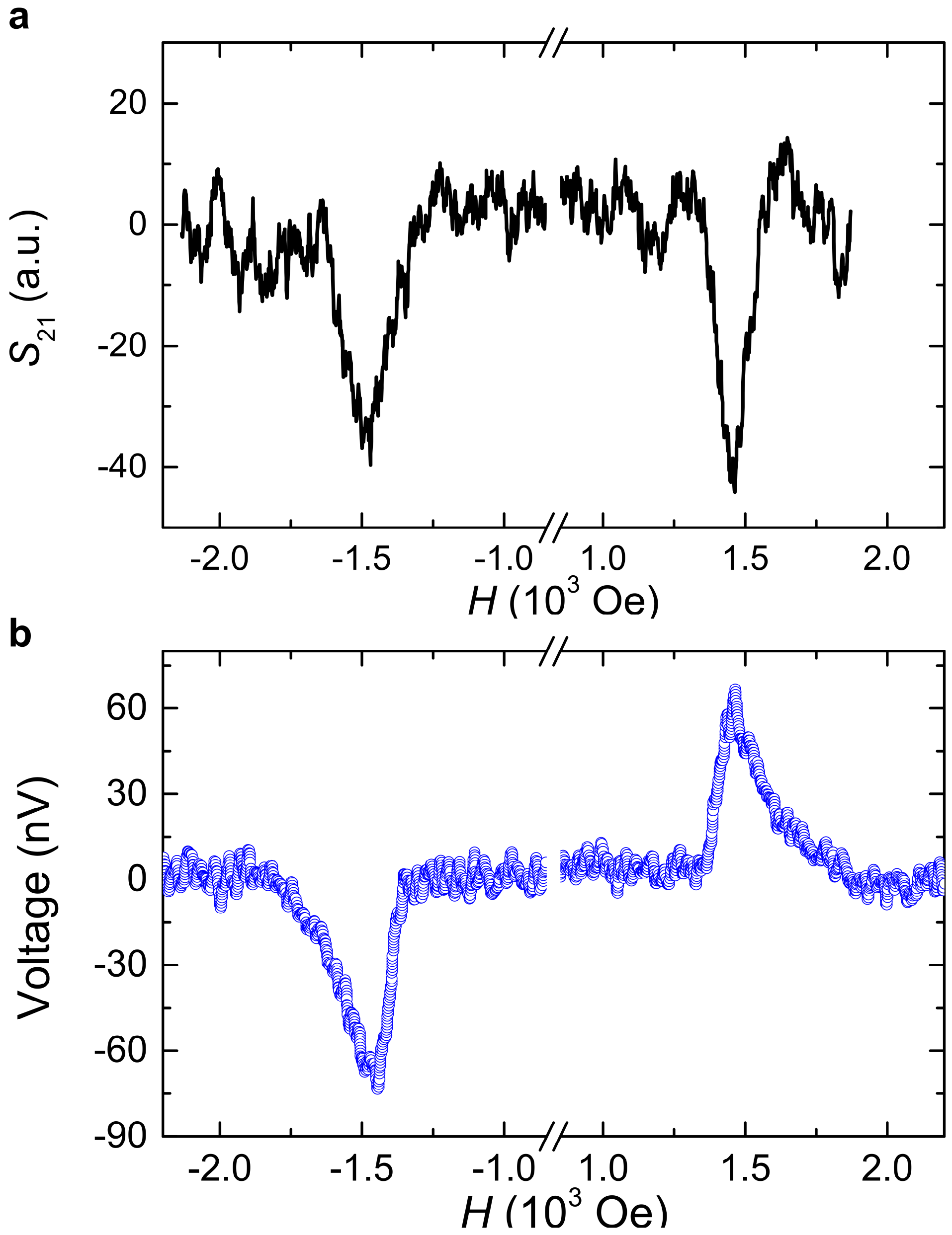

Supplementary Figure 1 | The measured voltage due to ferromagnetic resonance of Py. **a.** Ferromagnetic resonance spectra of the Py electrode deposited on the SmB_6 single crystal using a vector network analyzer with the microwave power of 1 mW. **b**. The measured voltage as a function of the magnetic field using a signal generator with the power of 200 mW. These results are obtained at 1.7 K with the microwave frequency of 11.3 GHz.

Supplementary Figure 2

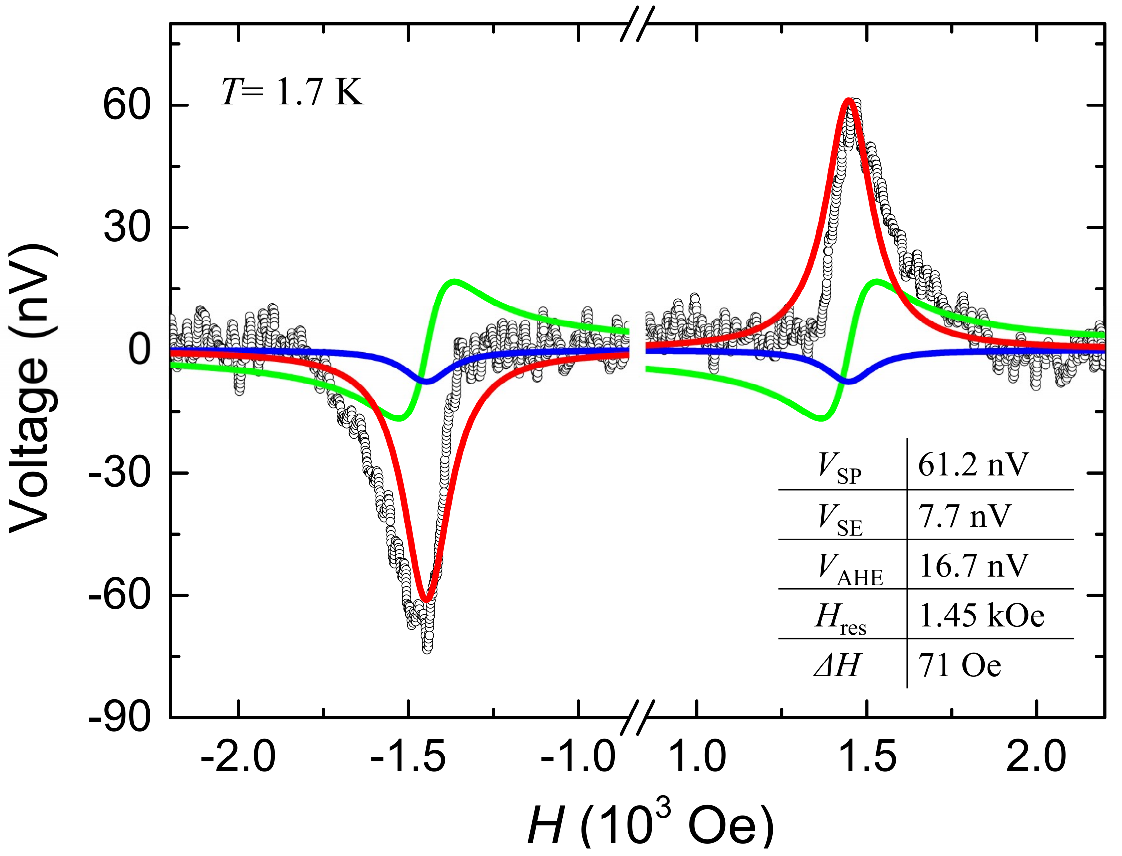

Supplementary Figure 2 | Numerical fitting for the measured voltage. The results are measured at 1.7 K with the microwave frequency of 11.3 GHz and power of 200 mW. The black circles, red lines, blue lines and green lines correspond to the original measured data, the voltages due to spin pumping and inverse Edelstein effect, the Seebeck effect, and the anomalous Hall effect, respectively. The inset table summarizes the parameters obtained from the numerical fitting procedure.

Supplementary Figure 3

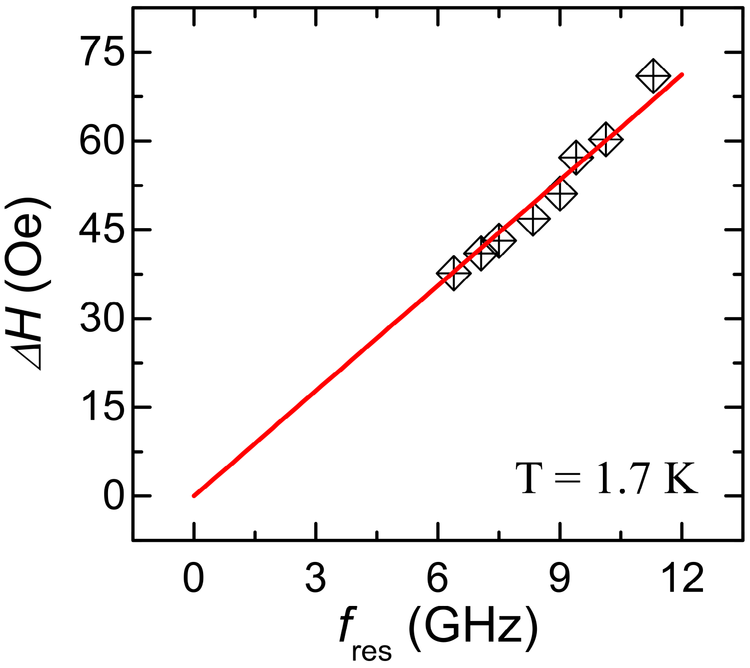

Supplementary Figure 3 | The half linewidth vs. the resonance microwave frequency. The results are measured at 1.7 K with the microwave power of 100 mW. The Gilbert damping can be obtained from the slope of the linearly fitted curve (red line).

Supplementary Figure 4

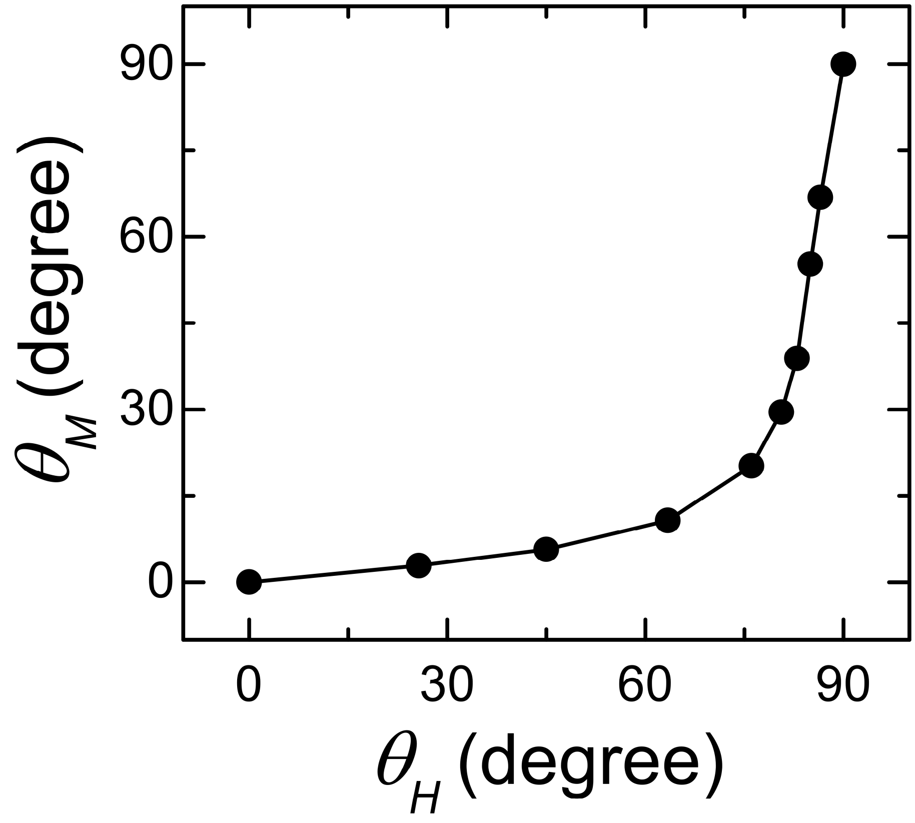

Supplementary Figure 4 | The magnetization angle of Py vs. the magnetic field angle. The Py magnetization angle is calculated from the resonance magnetic field vs. the magnetic field angle results.

Supplementary Figure 5

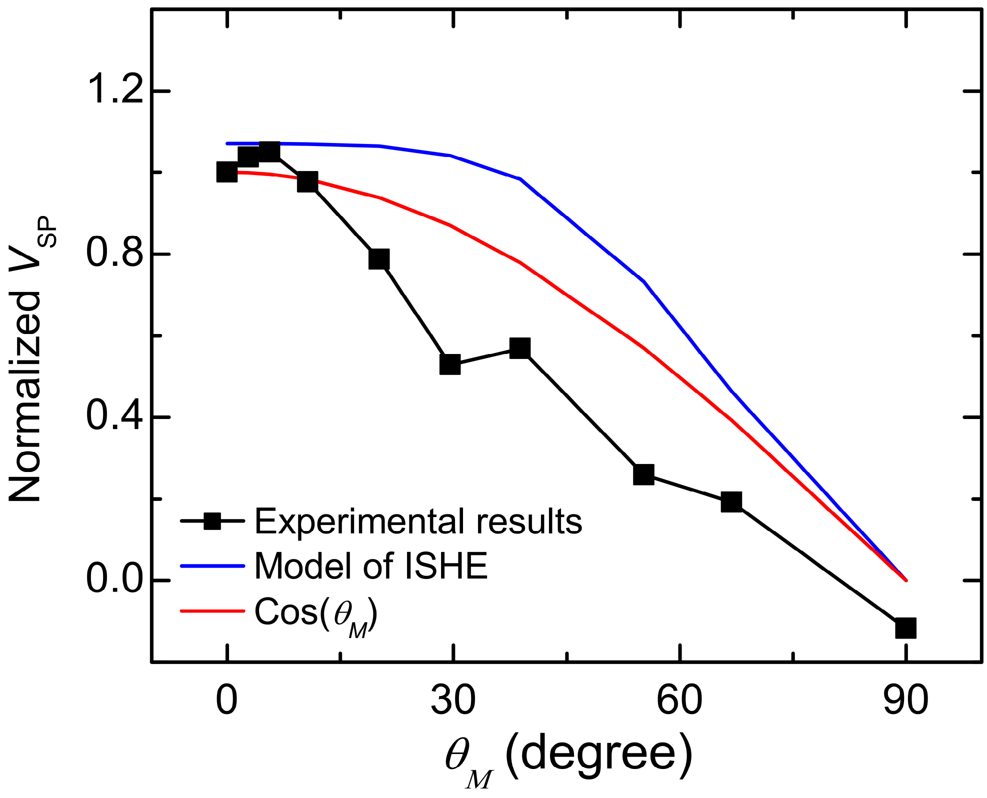

Supplementary Figure 5 | Magnetization angle dependence of the spin pumping voltage. The black squares, the blue line and the red line correspond to normalized experimental results, calculated results based on the model for spin pumping and inverse spin Hall effect (ISHE) and the Cosine function.

Supplementary Note 1. Ferromagnetic resonance of $Ni_{80}Fe_{20}$ and the spin pumping measurement.

The ferromagnetic resonance (FMR) of $Ni_{80}Fe_{20}$ (Py) is measured using the vector network analyzer (VNA, Agilent E5071C) connected with a coplanar waveguide under the Py. Supplementary Figure 1a shows a typical forward amplitude of the complex transmission coefficients (S_{21}) as a function of the magnetic field measured at 1.7 K with the microwave frequency of 11.3 GHz and power of 1 mW. In an external magnetic field, the magnetization of the Py dynamics could be described by the Landau-Lifshitz-Gilbert equation[1,2]:

$$\frac{d\mathbf{M}}{dt} = -\gamma \mathbf{M} \times \mathbf{H}_{\mathrm{eff}} + \frac{\alpha}{M_{\mathrm{S}}} \mathbf{M} \times \frac{d\mathbf{M}}{dt} \tag{1}$$

Where $\mathbf{M}$ is the magnetization vector, $\mathbf{H}_{\mathrm{eff}}$ is the total effective magnetic field, γ is the gyromagnetic ratio, $M_{\mathrm{S}} = |\mathbf{M}|$ is the saturation magnetization, and α is the Gilbert damping. At the resonance condition, the precessing magnetization of Py electrode absorbs the microwave, and as a result of which, the measured amplitude of the complex transmission coefficient shows a minimum. Away from the resonance condition, the Py electrode does not affect the transmission coefficient.

Due to the angular momentum transfer at the interface between Py and SmB_6[3,4], the precessing magnetization of the Py electrode gives rise to the spin injection into the surface states of the Kondo topological insulator SmB_6. To achieve a better signal to noise ratio for the measurement of spin pumping and inverse Edelstein effect, we replace the vector network analyzer with a signal generator to provide much higher microwave power. Under the same microwave frequency of 11.3 GHz but a much higher power of 200 mW, we observe a voltage signal at the magnetic fields

(Supplementary Figure 1b) that are the same as the resonance magnetic fields of the Py electrode. This feature confirms that the measured voltage is indeed a result from Py magnetization precessing. Furthermore, the measured voltage shows a much better signal to noise ratio compared to the FMR measurement of Py around the resonance magnetic fields (H_{res}). Hence, we use the voltage measurement to obtain the resonance magnetic fields and the half linewidths.

Supplementary Note 2. The numerical fitting procedure for the analysis of the measured voltage.

There are three contributions to the measured voltage around the resonance magnetic fields. The major one is due to the inverse Edelstein effect of the spin polarization in the topological surface states (V_{SP}), which is expected to exhibit a symmetric Lorentzian shape. When the magnetization of Py switches its direction, the voltage will change sign as well due to the spin injection with the opposite polarization. Hence, the measured voltage is either negative or positive depending on the direction of the Py magnetization or the magnetic field. The second one is due to the Seebeck effect (V_{SE}), which is also expected to exhibit a symmetric Lorentzian shape. The origin of this effect is related to the heating via the absorption of the microwave because of a possible small discrepancy in the sample position from the center of the coplanar waveguide. Thus, no matter in positive or negative magnetic field, the sign of the voltage from Seebeck effect should not change at all. The last one is due to the anomalous Hall effect (V_{AHE}), which arises from the Py layer in the presence of an in-plane part of the microwave electric field[5]. This effect shows an asymmetric Lorentzian shape. To obtain the contribution ratio of these three contributions, we numerically fit the magnetic field (H) dependence of the voltage curves. The fitting procedure is described as follows using the measured curve in Supplementary Figure 1b as an example.

First, the measured curve is fitted by the following equation:

$$V(H) = V_{\mathrm{S}} \frac{\Delta H^2}{(H - H_{\mathrm{res}})^2 + \Delta H^2} + V_{\mathrm{AS}} \frac{-2\Delta H (H - H_{\mathrm{res}})}{(H - H_{\mathrm{res}})^2 + \Delta H^2} \tag{2}$$

where V_{S} and V_{AS} are the voltage amplitudes for the symmetric and asymmetric Lorentzian components, respectively, and ΔH is the half-line width. Since the V_{AHE} is the only contribution to the asymmetric component, V_{AHE} is equal to V_{AS}. For V_{SP} and V_{SE}, they both show symmetric Lorentzian shape but with different sign for positive and negative magnetic fields. Hence, we can calculate each of them from the results of $V_{\mathrm{S}}(+)$ and $V_{\mathrm{S}}(-)$ obtained for the positive and negative H, respectively.

$$V_{\mathrm{SP}} = \frac{V_{\mathrm{S}}(+) - V_{\mathrm{S}}(-)}{2} \tag{3}$$

$$V_{\mathrm{SE}} = \frac{V_{\mathrm{S}}(+) + V_{\mathrm{S}}(-)}{2} \tag{4}$$

As shown in Supplementary Figure 2, the black circles indicate the measured voltage. The red, blue, and green solid lines correspond to the numerical fitted components due to the spin pumping and inverse Edelstein effect, the spin Seebeck effect, and the anomalous Hall effect, respectively. And the obtained parameters are summarized in the inset table.

Supplementary Note 3. Estimation of the spin mixing conductance

The half-line width vs. the excitation microwave frequency (f_{res}) obtained from the magnetic field dependence of the voltage under various microwave frequency is summarized in Supplementary Figure 3. These results are measured at 1.7 K with the microwave power of 100

mW (black squares). The Gilbert damping is calculated to be 0.0166 ± 0.0006 obtained from slope of the linearly fitted curve (red lines).

$$\alpha = (\frac{\gamma}{2\pi})\frac{\partial(\Delta H)}{\partial(f_{\mathrm{res}})} \tag{5}$$

in which γ is the geomagnetic ratio. This value is considerably larger than the value of 0.0057 ± 0.0005 measured on 20 nm Py grown on Si/SiO_2 substrate at 2 K[6]. The interfacial effective spin mixing conductance (G_{eff}) is estimated to be (1.0 ± 0.1) × 10^{20} m^{-2} by comparing the Gilbert damping of Py on SmB_6 and SiO_2 based on the following equation:

$$G_{\mathrm{eff}} = \frac{4\pi M_{\mathrm{S}} t}{g\mu_{\mathrm{B}}}(\alpha_{Py/SmB_6} - \alpha_{Py/SiO_2}) \tag{6}$$

where μ_{B} is the Bohr magneton, t and M_{S} are the thickness and magnetization of the of the Py film. However, we should note that besides the spin pumping, the Gilbert damping can also be largely affected by some other factors, like sample quality and surface roughness. Therefore, the quantitative extraction of the values for the injected spin current and the effective spin-to-charge conversion efficiency need further studies.

Supplementary Note 4. The magnetization angle dependence of the spin pumping voltage

As shown in Fig. 5 in our manuscript, the spin pumping voltage exhibits a linear relationship as the function of the magnetization angle. This linear relationship cannot be explained at all by the model based on the spin pumping and inverse spin Hall effect done by Ando *et al*[5] (blue line in Supplementary Figure 5). Besides, the Cosine function also fails to describe our results (red line

in Supplementary Figure 5). We believe that the complete understanding of the V_{SP} as a function of the θ_M needs further theoretical studies.

Supplementary References:

1 Landau, L. & Lifshitz, E. On the theory of the dispersion of magnetic permeability in ferromagnetic bodies. *Phys. Z. Sowjetunion* **8**, 153, (1935).

2 Gilbert, T. L. A phenomenological theory of damping in ferromagnetic materials. *Magnetics, IEEE Transactions on* **40**, 3443-3449, (2004).

3 Tserkovnyak, Y., Brataas, A. & Bauer, G. E. W. Spin pumping and magnetization dynamics in metallic multilayers. *Phys. Rev. B* **66**, 224403, (2002).

4 Tserkovnyak, Y., Brataas, A., Bauer, G. E. W. & Halperin, B. I. Nonlocal magnetization dynamics in ferromagnetic heterostructures. *Rev. Mod. Phys.* **77**, 1375-1421, (2005).

5 Ando, K. *et al.* Inverse spin-Hall effect induced by spin pumping in metallic system. *J. Appl. Phys.* **109**, 103913, (2011).

6 Zhao, Y. *et al.* Experimental Investigation of Temperature-Dependent Gilbert Damping in Permalloy Thin Films. *Scientific Reports* **6**, 22890, (2016).
